# The association between alcohol consumption and sleep disorders among older people in the general population

**DOI:** 10.1038/s41598-020-62227-0

**Published:** 2020-03-24

**Authors:** Annie Britton, Linda Ng Fat, Aidan Neligan

**Affiliations:** 1 Department of Epidemiology and Public Health, University College London, London, UK; 2 Department of Neurology, Homerton University Hospital NHS Foundation Trust, London, UK; 3 Department of Clinical and Experimental Epilepsy, UCL Queen Square Institute of Neurology, London, UK

**Keywords:** Medical research, Risk factors

## Abstract

The relationship between alcohol consumption and sleep disturbance is complex. The association of alcohol dependence with insomnia is likely to be bidirectional in nature. Alcohol use is common among older people in many societies and the prevalence of insomnia tends to increase with age, therefore this group warrants particular consideration. We explored the cross sectional and long term (30 years) associations between alcohol drinking (volume and hazardous drinking) and sleep duration and insomnia in a general population study of older adults (6,117 male and female civil servants followed for 30 years). For men, drinking more than 21 units (approximately 168 grams) of alcohol per week, compared with not drinking, was associated with waking several times a night (odds ratio 1.30, confidence intervals 1.02–1.66). Men who maintained a heavy volume of drinking over the three decades of observation, or who had an unstable consumption pattern, tended to have worse sleep profiles in terms of waking tired and waking several times. Sustained male hazardous drinking (as measured by the AUDIT-C scale) was also associated with worse sleep profiles. Findings for women were not so clear. In this population based setting, drinking high volumes of alcohol may contribute to the prevalence of sleep problems in older age, particularly for men. People in this age group should be discouraged from using alcohol as a sleep aid.

## Introduction

Inadequate sleep is estimated to affect about one in five adults^1^. Insomnia symptoms (short sleep and disturbed sleep) are associated with an increased risk of a range of chronic health conditions, such as diabetes^2^, hypertension^3^ and all-cause mortality^4^.

The relationship between alcohol consumption and sleep disturbance is complex. Alcohol acts as a sedative and reduces sleep onset latency^5^, and as such, may be used proactively to relieve insomnia^6^. However, there is evidence that alcohol consumption also disrupts sleep, particularly the period of rapid eye movement (REM) sleep^7^. The perpetual use of alcohol as a sleep aid may be a counterproductive long-term strategy as alcohol disrupts sleep quality and intensifies the need to consume more alcohol^8^. The association of alcohol dependence with insomnia may be bidirectional in nature^9^.

Heavy consumption of alcohol over an extended period of time leads to increased tolerance and this tolerance is accompanied by adaptation of the neurotransmitter systems^5^. Furthermore, long-term consequences of alcohol may lead to changes in sleep regulation. The influence of alcohol on sleep therefore needs to be evaluated by exploring both the short term effects on sleep (cross sectional data) and the long-term consequences (longitudinal data of repeated measures). At present, most literature is based on cross-sectional studies and thus cannot assess direction of effects^6^.

Alcohol consumption among the elderly has increased^10^ and the prevalence of insomnia tends to increase with age^11^, therefore this age group warrants particular consideration.

This paper will address the following aims: (1) to explore the cross-sectional association between alcohol drinking and sleep problems in a general population study of older adults and (2) to explore the long term association between typologies of alcohol drinking and chronic sleep problems. To our knowledge, this is the first paper to utilize individual longitudinal repeat data on sleep and alcohol in this way.

## Methods

The Whitehall II study was established in 1985 as a longitudinal study to examine the socioeconomic gradient in health and disease among 10,308 civil servants (6895 men and 3413 women)^12^. All civil servants aged 35–55 years in 20 London-based departments were invited to participate by letter and 73% agreed. Baseline examination (Phase 1) took place during 1985–1988 and involved a clinical examination and a self-administered questionnaire containing sections on demographic characteristics, health, lifestyle factors, work characteristics, social support and life events. Subsequent phases of data collection have alternated between postal questionnaire alone and postal questionnaire accompanied by a clinical examination.

The University College London Medical School Committee on the ethics of human research approved the Whitehall II study all research was performed in accordance with relevant guidelines/regulations. Written informed consent was obtained at baseline and renewed at each contact. Whitehall II data, protocols, and other metadata are available to bona fide researchers for research purposes. (Data sharing policy is available at http://www.ucl.ac.uk/whitehallII/data-sharing).

During three decades of follow-up, repeated measures were obtained via a self-completed questionnaire of insomnia symptoms and sleep duration and repeated measures of alcohol consumption and problem drinking.

### Assessment of Sleep measures

Sleep duration was assessed at phases 1 (1985–88), 5 (1997–99), 7 (2002–04, 9 (2007–09), and 11 (2012–13) by asking participants: “how many hours of sleep do you have on an average week night?” Respondent choices were: 5 hours or less, 6 hours, 7 hours, 8 hours, and 9 hours or more”.

Sleep disturbances were assessed at phases 5, 7, 9, and 11 using the 4-item Jenkins Scale^13^. This scale includes 4 questions on “having trouble falling asleep”, “waking up several times per night,” “having trouble staying asleep,” “waking up after the usual amount of sleep feeling tired and worn out” (i.e., waking without feeling refreshed) over past 30 days; all items have a 6-point response scale (1 = never; 2 = 1–3 days; 3 = 4–7 days; 4 = 8–14 days; 5 = 15–21 days; 6 = 22–30 days).

All sleep variables were dichotomized to form groups that were, as closely as possible, similar in size in relation to the total sample. These were as follows; Sleep duration (<7 versus 7+ hours (reference)), Trouble staying asleep over 30 days (4+ v. <4 days), Trouble falling asleep (1+ v. 0 days), Wake as usual but tired (1+ v. 0 days), Wake several times a night, (4+ v. <4 days). Dichotomization in this way allowed for a large enough sample to use in statistical analyses when stratifying by men and women. For all sleep variables the sleep category corresponding to better sleep was treated as the reference category.

Chronic sleep problems were defined as those when participants who reported a sleep problem (based on the above dichotomy) at three or more data collection phases over the follow-up period.

### Assessment of alcohol consumption

#### Volume of consumption (phases 1, 3, 5, 7, 9, 11)

Participants were asked to report the number of alcoholic drinks they had consumed in the last 7 days. Drinks were converted into UK units of alcohol (whereby one unit is equivalent to 8 g of ethanol) using a conservative estimate of one UK unit for each measure of spirits and glass of wine, and two UK units for each pint of beer. These converted measurements were then summed to define the total weekly number of UK units consumed. Participants who did not drink alcohol in the past year were classified as ‘non-drinkers’.

#### Retrospective alcohol life-course grid

Life course alcohol consumption was defined using decade based grids^14^ (Appendix 1) starting with information in the teens (16–19 years) and spanning to the eighties (and older) on the three components of the AUDIT-C questionnaire: frequency of consumption, number of drinks on a typical drinking day, and frequency of consuming six or more drinks in a single occasion. AUDIT-C cases were defined as those scoring 5 or more points^15^. Non-drinkers (participants who did not drink alcohol in the previous year) were excluded from the classification (Appendix 2)

#### Alcohol typologies based on volume

Typologies of alcohol consumption over the measurement periods were then created^16^: (1) Stable None, (2) Stable moderate, (3) Stable heavy, (4) Unstable moderate (at least half of the phases were moderate), (5) Unstable heavy and (6) Former drinkers (previously reported consumption but none in the most recent phase). “Moderate” (within UK guidelines^17^ (1–14 [8–112 g] units per week), and “Heavy” (above guidelines (15 + units). When an individual reported moderate and heavy on an equal number of occasions, participants were assigned to the unstable heavy drinking group. There were 44 participants who did not fall into either of the categories and were excluded.

#### Hazardous drinking over follow-up

Participants were considered to be chronically hazardous drinkers if they were AUDIT-C positive on three of more data collection phases (in the retrospective alcohol life-course grid).

#### Analyses

Cross-sectional associations were explored using phase 11 data. Longitudinal associations used data from phases 1 through to 11. Logistic regression analyses with the sleep variables as the outcome variable, and alcohol variables as the main exposure, were performed in Stata v15, adjusting for age. Models were carried out separately for the different alcohol measurements, and were stratified by men and women.

## Results

In 2012–2013, 70.9% of the original cohort who were still alive (age range 61–81 years), participated in phase 11. Of these 6,318 men and women, 6117 (96.8%) had data on alcohol and sleep. Men consumed more alcohol than women with 15.7% consuming 21 or more units per week compared to only 2.4% of women (Table 1). 30.5% men and 12.8% women scored more than 5 on the AUDIT score, indicating hazardous drinking.

**Table 1:** Characteristics of Whitehall II sample at Phase 11 (2012–2013).

	Men n (%)	Women n (%)	Chi-squared test
	4334	1783	
Mean age (years)	69.4	69.6	
***Alcohol volume per week***			
Non-drinker^a^	263 (6.1)	287 (16.1)	
None in the past week	441 (10.2)	351 (19.7)	
1–14 units^b^	2384 (55.0)	999 (56.0)	
14–21 units	566 (13.1)	103 (5.8)	
21+ units	680 (15.7)	43 (2.4)	P < 0.001
**AUDIT case-ness (>5)**^**c**^			
No	2872 (69.5)	1322 (87.2)	
Yes	1,258 (30.5)	194 (12.8)	P < 0.001
***Sleep duration (hours per night)***			
<7	2797 (63.7)	980 (54.4)	
7+	1596 (36.3)	823 (45.6)	P < 0.001
***Trouble staying asleep (days per month)***			
None/1–3 days	2659 (60.9)	1003 (55.7)	
4+ days	1708 (39.1)	797 (44.3)	P < 0.001
***Trouble falling asleep (days per month)***			
None	2212 (50.5)	547 (30.4)	
1+ days	2168 (49.5)	1250 (69.6)	P < 0.001
***Wake as usual but tired (days per month)***			
None	2108 (48.2)	740 (41.1)	
1+ days	2269 (51.8)	1059 (58.9)	P < 0.001
***Wake several times a night (days/month)***			
None/1–3 days	1867 (42.7)	731 (40.8)	
4+ days	2503 (57.3)	1060 (59.2)	P = 0.168

^a^Non-drinker defined as not having a drink in the past year.

^b^1 unit = 8 grams of alcohol.

^c^Excludes non-drinkers.

The most common drinking typologies over the three decades of observation were stable moderate drinkers (21.2%) and unstable moderate (29.2%). Women were more likely to report being former drinkers than men (24.8% and 12.8% respectively) (Table 2). Chronic hazardous drinking was indicated in 38% men and 17% women.

**Table 2:** Alcohol typologies and chronic sleep problems (reported at 3 or more phases) over 30 years follow up.

	Men n (%)	Women n (%)	Chi-squared test
***Volume alcohol***			
Stable none	100 (3.0)	111 (8.7)	
Stable moderate^a^	682 (20.3)	299 (23.6)	
Stable heavy^b^	409 (12.2)	25 (2.0)	
Unstable moderate	956 (28.4)	396 (31.2)	
Unstable heavy	790 (23.5)	122 (9.6)	
Former drinkers	430 (12.8)	315 (24.8)	P < 0.001
***Hazardous drinking***			
Not chronic AUDIT	2238 (61.3)	1174 (83.3)	
Audit positive at 3+ phases	1414 (38.7)	235 (16.7)	P < 0.001
***Sleep duration***			
<7 hours	2465 (67.5)	855 (60.7)	
7+ hours	1187 (32.5)	554 (39.3)	P < 0.001
***Trouble staying asleep (days per month)***			
None/1–3 days	2672 (73.2)	924 (65.6)	
4+ days	934 (25.9)	451 (32.8)	P < 0.001
***Trouble falling asleep (days per month)***			
None	2210 (60.5)	574 (40.7)	
1+ days	1435 (39.4)	817 (58.7)	P < 0.001
***Wake as usual but tired (days per month)***			
None	2068 (56.6)	629 (44.6)	
1+ days	1556 (42.9)	699 (52.6)	P < 0.001
***Wake several times a night (days per month)***			
None/1–3 days	2077 (56.9)	672 (47.7)	
4+ days	1513 (42.1)	699 (51.0)	P < 0.001

^a^Moderate = 1–14 units.

^b^Heavy = 14+ units.

In terms of sleep problems, men were more likely to report sleeping less than 7 hours per night than women (63.7% men compared to 54.4% women). However, women were more likely to report trouble falling asleep (69.6% compared to 49.5% men) (Table 1). Over the thirty years follow up, women generally reported more chronic sleep problems than men (Table 2). More than half the women studied reported trouble falling asleep, waking tired, and/or waking several times a night.

Cross sectional analyses between alcohol (both volume and hazardous) and sleep problems found that men drinking more than 21 units per week or drinking hazardously were more likely to have disturbed sleep parameters than those not drinking in the past week or not drinking hazardously (Table 3). For example, men drinking 21+ units were more likely to wake several times a night than non-drinkers (OR 1.30 CI:1.02–1.66). For women, the picture is less clear. There is a suggestion that those women drinking more than 21 units were less likely to have short sleep (less than 7 hours) compared to non-drinkers (OR 0.39 CI: 0.19–0.81).

**Table 3:** Cross-sectional association between alcohol and sleep problems (adjusted for age).

	MEN		WOMEN	
	OR	95% CI	OR	95% CI
***Sleep duration (***<***7*** ***hours compared to 7*** + ***hours)***				
No alcohol in the past week^a^	1		1	
Non-drinker^b^	1.22	0.89–1.67	1.01	0.74–1.38
1–14 units^c^	0.97	0.79–1.20	0.81	0.64–1.04
14–21 units	0.92	0.71–1.20	0.68	0.44–1.07
21+ units	1.15	0.90–1.47	0.39	0.19–0.81
AUDIT non-case	1		1	
AUDIT Case	1.08	0.94–1.24	0.68	0.50–0.93
***Trouble staying asleep (4*** + ***vs*** < ***4 days per month)***				
No alcohol in the past week	1		1	
Non-drinker	1.05	0.77–1.45	1.05	0.77–1.44
1–14 units	1.07	0.87–1.32	0.92	0.72–1.18
14–21 units	1.23	0.95–1.59	0.95	0.61–1.48
21+ units	1.26	0.98–1.62	0.96	0.50–1.82
Audit non-Case	1		1	
AUDIT Case	1.22	1.06–1.40	1.03	0.76–1.39
***Trouble falling asleep (1*** + ***vs 0 days per month)***				
No alcohol in the past week	1		1	
Non-drinker	1.15	0.85–1.57	0.89	0.63–1.24
1–14 units	1.22	0.99–1.50	0.96	0.73–1.26
14–21 units	1.45	1.13–1.86	0.67	0.42–1.05
21+ units	1.11	0.87–1.41	0.64	0.33–1.25
AUDIT non-case	1		1	
AUDIT Case	0.98	0.85–1.12	0.85	0.61–1.17
***Wake tired (1*** + ***vs 0 days per month)***				
No alcohol in the past week	1		1	
Non-drinker	1.02	0.75–1.39	1.00	0.72–1.38
1–14 units	0.95	0.78–1.17	0.87	0.68–1.12
14–21 units	1.06	0.83–1.37	0.88	0.56–1.37
21+ units	1.02	0.80–1.30	0.58	0.30–1.10
AUDIT non-case	1		1	
AUDIT Case	1.04	0.91–1.19	0.89	0.65–1.21
***Wake several times (4*** + ***vs*** < ***4 days per month)***				
No alcohol in the past week	1		1	
Non-drinker	0.90	0.66–1.23	0.96	0.70–1.32
1–14 units	1.08	0.88–1.33	1.13	0.88–1.45
14–21 units	1.32	1.02–1.70	1.13	0.72–1.77
21+ units	1.30	1.02–1.66	1.29	0.66–2.52
AUDIT non-case	1		1	
AUDIT Case	1.19	1.04–1.37	1.23	0.90–1.69

^a^Excludes non-drinkers.

^b^Non-drinker defined as not having a drink in the past year.

^c^1 unit = 8 grams of alcohol.

The relationship between longitudinal alcohol typologies and chronic sleep problems reflected the cross-sectional picture (Table 4). For men, compared to stable moderate drinkers, those who were stable heavy drinkers were more likely to wake tired (RRR 1.37 CI: 1.02–1.84) and wake several times a night (RRR 1.52 CI: 1.13–2.05). For women there were less clear risks associated between drinking and sleep problems.

**Table 4:** Alcohol typologies and chronic sleep problems (reported on three or more phases) over 30 years follow up (adjusted for age).

	RRR	95% CI	RRR	95% CI
***Sleep duration (*<*5*** ***hours compared to 5*** + ***hours)***				
Stable none	1.22	0.78–1.92	1.06	0.63–1.77
Stable moderate^a^	1		1	
Stable heavy^b^	0.82	0.64–1.06	0.37	0.16–0.85
Unstable moderate	1.11	0.91–1.37	0.98	0.69–1.39
Unstable heavy	1.18	0.95–1.46	0.67	0.42–1.06
Former drinkers	0.96	0.75–1.23	0.97	0.67–1.40
Always AUDIT non-case	1		1	
Case on 3+ occasions	1.21	1.05–1.39	0.62	0.46–0.84
***Trouble staying asleep (15*** + ***days vs*** < ***15 days per month)***				
Stable none	0.84	0.55–1.29	1.18	0.73–1.92
Stable moderate	1		1	
Stable heavy	1.42	1.10–1.83	1.16	0.47–2.85
Unstable moderate	1.18	0.97–1.44	1.06	0.76–1.48
Unstable heavy	1.45	1.17–1.79	1.09	0.68–1.73
Former drinkers	1.10	0.86–1.41	0.97	0.68–1.38
Always Audit non-Case	1		1	
Case on 3+ occasions	1.47	1.28–1.69	1.31	0.94–1.81
***Trouble falling asleep (15*** + ***vs*** < ***15 days per month)***				
Stable none	0.96	0.60–1.53	0.76	0.37–1.53
Stable moderate	1		1	
Stable heavy	1.06	0.80–1.40	0.38	0.13–1.10
Unstable moderate	1.05	0.84–1.31	0.65	0.40–1.08
Unstable heavy	1.25	0.98–1.58	0.47	0.25–0.86
Former drinkers	0.90	0.69–1.18	0.89	0.52–1.55
Always Audit non-Case	1		1	
Case on 3+ occasions	1.14	0.98–1.33	0.68	0.45–1.04
***Wake tired (15*** + ***vs*** < ***15 days per month)***				
Stable none	0.84	0.53–1.32	1.08	0.58–1.99
Stable moderate	1		1	
Stable heavy	1.37	1.02–1.84	0.48	0.18–1.30
Unstable moderate	1.38	1.10–1.73	1.08	0.70–1.66
Unstable heavy	1.41	1.11–1.79	0.61	0.36–1.05
Former drinkers	1.31	0.99–1.73	0.93	0.60–1.44
Always AUDIT non-case	1		1	
Case on 3+ occasions	1.43	1.21–1.68	1.31	0.84–2.03
***Wake several times (15*** + ***vs*** < ***15 days per month)***				
Stable none	0.72	0.45–1.13	1.33	0.71–2.48
Stable moderate	1		1	
Stable heavy	1.52	1.13–2.05	1.48	0.42–5.17
Unstable moderate	1.40	1.11–1.77	1.21	0.80–1.82
Unstable heavy	1.61	1.26–2.07	0.84	0.49–1.45
Former drinkers	1.16	0.87–1.53	1.01	0.66–1.56
Always Audit non-Case	1		1	
Case on 3+ occasions	1.42	1.20–1.68	1.22	0.81–1.83

^a^Moderate = 1–14 units.

^b^Heavy = 14+ units.

## Discussion

In this large, population based study of older adults, we found that, for men, drinking more than 21 units per week, compared with not drinking, was associated with disturbed sleep (cross sectional analyses). Those who maintained this heavy volume of drinking over the three decades of observation, or who drank in a potentially hazardous pattern tended to have worse sleep profiles in terms waking tired and waking several times. The findings were not so clear among women and the reasons for sex differences warrants further research.

In a recent cross-sectional population study of 187,950 adults in the United States short sleep prevalence was higher among adults who consumed any alcohol compared with those who never consume alcohol^18^. Our findings contrast with this study in that we did not find strong association between drinking and sleep duration. The disparity may, in part, be due to ethnic differences. Jackson *et al*. note that the prevalence of short sleep across alcohol consumption patterns was more variable among whites, and the majority of Whitehall II participants are white.

There are few other longitudinal population based studies with which to compare our findings. Most are based on alcoholics in clinic settings^19^. Among 1,920 community dwelling men and women, those with persistent alcohol dependence had higher odds of insomnia that those without alcohol dependence over a fifteen year follow-up^20^. Whilst we did not measure alcohol dependence, we did find such an association between hazardous drinking and disturbed sleep in our data.

Our finding that those who have trouble falling asleep were more likely to be persistent heavy drinking suggests that they may be using alcohol as a sedative. This is partially corroborated in an earlier study on same population. The Whitehall II participants were asked about reasons for change in drinking over the last 10 years and an increase in consumption was cited as a means to help get to sleep was by 6% of men and 5% of women^21^.

Our study has limitations. For instance, we used self-reported alcohol consumption data and self-reported sleep data and therefore these measures may be at risk of reporting bias. The population may not be representative of all older adults in the UK and it is unlikely that the full spectrum of drinking behavior is represented. However, the mean consumption is similar to that reported in representative studies, such as Health Survey for England and English Longitudinal study of Ageing^22^. Another limitation is that we were not able to capture fully patterns of consumption in terms of binging. Drinking appears to have differential effects on sleep depending on chronic versus acute dosage^18^ Our cut-offs for drinking exposures and sleep problems are largely arbitrary and it is possible to other subtle relationships are masked. Findings using self-reported sleep can only support the hypothesis that alcohol impacts on underlying sleep architecture (for example reduction or suppression of REM sleep), which would need to be confirmed by overnight polysomnography sleep studies. Despite these limitations, this study has important strengths. The repeated collection of alcohol and sleep data over such a long period is unique. We were thus able to look at long-term drinking typologies and persistent sleep problems over three decades.

## Conclusion

In this population based setting, drinking high volumes of alcohol or drinking hazardously may contribute to the prevalence of sleep problems in older age. Those with disrupted sleep should consider reducing alcohol consumption and people in this age group, particularly men, should be discouraged from using alcohol as a sleep aid. It is well recognized that sleep problems have a significant impact on quality of life with increased morbidity and mortality seen in population studies^3^. Identifying people at risk of sleep disturbances as a result of their drinking may have important public health benefits.

## Supplementary information

Appendix 1 and 2.
