# Supplementary material for: The association between alcohol consumption and sleep disorders among older people in the general population
Source: Sci Rep. 2020 Mar 24;10:5275. doi: 10.1038/s41598-020-62227-0 (PMC7093458; doi:10.1038/s41598-020-62227-0)

**The association between alcohol consumption and sleep disorders among older people in the general population**

Annie Britton PhD^1*^, Linda Ng Fat PhD^2^ , Aidan Neligan PhD MRCP^3,4^

^1,2^ Department of Epidemiology and Public Health, University College London, London UK

^3^ Department of Neurology, Homerton University Hospital NHS Foundation Trust, London UK

^4^ Department of Clinical and Experimental Epilepsy, UCL Queen Square Institute of Neurology, London, UK

Appendix 1: Lifetime alcohol questionnaire used at Phase 11 of the Whitehall II study


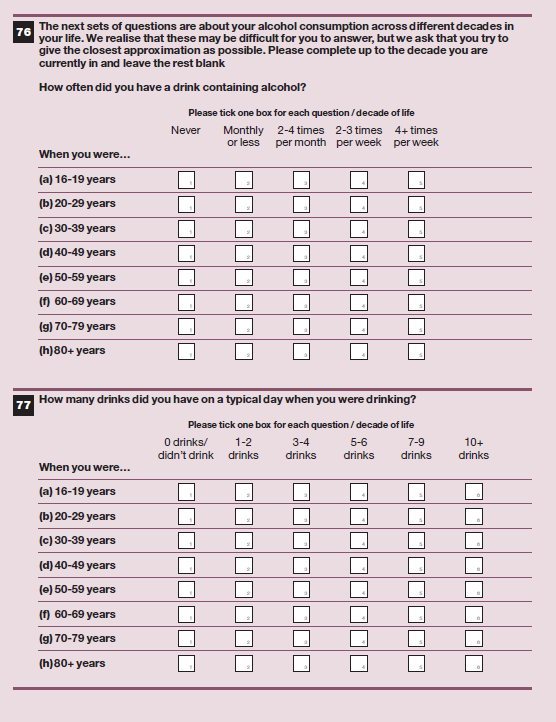


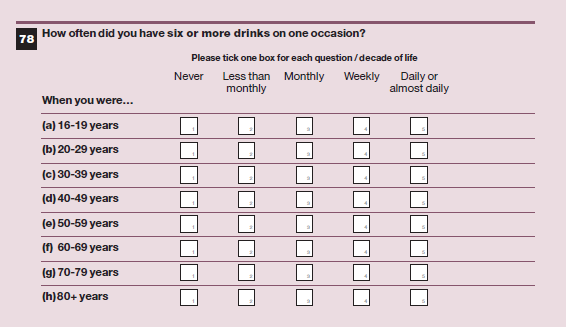


Appendix 2 AUDIT-C scoring system


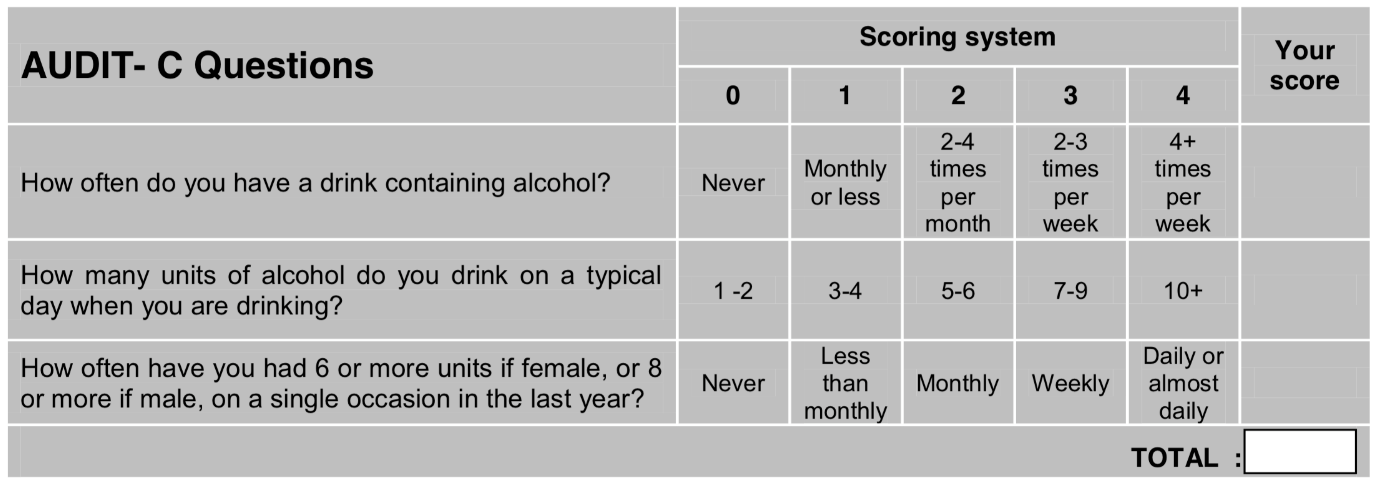

Supplement: Supplementary file 1 — Appendix 1 and 2. [file 41598_2020_62227_MOESM1_ESM.docx]
